# Mirror-Symmetry-Breaking in Poly[(9,9-di-*n*-octylfluorenyl- 2,7-diyl)-*alt*-biphenyl] (PF8P2) is Susceptible to Terpene Chirality, Achiral Solvents, and Mechanical Stirring

**DOI:** 10.3390/molecules18067035

**Published:** 2013-06-17

**Authors:** Michiya Fujiki, Yoshifumi Kawagoe, Yoko Nakano, Ayako Nakao

**Affiliations:** 1 Graduate School of Materials Science, Nara Institute of Science and Technology, 8916-5 Takayama, Ikoma, Nara 630-0192, Japan; E-Mails: y.nakano@berkeley.edu (Y.K.); n-ayako@ms.naist.jp (A.N.); 2 Department of Materials Science and Engineering, University of California, Berkeley, CA 94720-1760, USA; E-Mail: y.nakano@berkeley.edu

**Keywords:** limonene, mirror symmetry breaking, polymer, chirality, optically active, circular dichroism, circularly polarized luminescence, vortex, parity violation, homochirality

## Abstract

Solvent chirality transfer of (*S*)-/(*R*)-limonenes allows the instant generation of optically active **PF8P2** aggregates with distinct circular dichroism (CD)/circularly polarized luminescence (CPL) amplitudes with a high quantum yield of 16–20%. The present paper also reports subtle mirror-symmetry-breaking effects in CD-/CPL-amplitude and sign, CD/UV-vis spectral wavelengths, and photodynamics of the aggregates, though the reasons for the anomaly are unsolved. However, these photophysical properties depend on (i) the chemical natures of chiral and achiral molecules when used in solvent quantity, (ii) clockwise and counterclockwise stirring operations, and (iii) the order of addition of limonene and methanol to the chloroform solution.

## 1. Introduction

Mirror-symmetry-breaking (MSB) has long attracted scientists from diverse disciplines [1,2,3,4,5,6,7,8]. So far, several scenarios for efficient MSB have been demonstrated, such as Frank’s bifurcation-type MSB crystallizations and aggregation under seeded, spontaneous, and stochastic resonance conditions [9,10,11,12,13,14,15,16,17], autocatalytic asymmetric catalysis reactions [8,18,19], subjection to an intense static magnetic field with unpolarized light [20], vortex flow in a fluidic medium [21,22,23,24,25], spin-polarized electron beam [26], circularly polarized light irradiation [27,28,29,30,31,32,33], molecular chirality transfer to aggregation and film states at liquid-liquid, liquid crystalline-gas and gas-solid interfaces [34,35,36,37,38,39,40,41,42,43,44,45,46], mechanochemical force [47,48], and parity-violating weak nuclear force mediated by *Z*° boson [49,50,51,52,53,54,55,56,57,58,59,60,61,62,63,64,65,66,67,68,69,70,71]. Among these asymmetric syntheses, solvent chirality transfer operating in the liquid phase allows the instant generation of optically active species in an isotropic solution and as aggregates in a heterogeneous solution without catalysts at room temperature when appropriate optically inactive and/or achiral substances are chosen [72].

Early research on solvent chirality transfer focused on several molecules and polymers in isotropic solutions. Mason* et al.* reported the first induced circular dichroism (ICD) spectrum at *d-d* transitions of [Co(NH_3_)_6_](ClO_4_)_3_ by coordinating with diethyl-(+)-tartrate in aqueous solution [73]. Bosnich was the first to find ICD effects at n-π* transitions of aromatic ketones including benzil and benzophenone in (*S*,*S*)-2,3-butanediol [74]. Hayward* et al.* studied the ICD effects of aliphatic ketones in chiral tetrahydrofuranols [75]. Noack suggested the existence of a molecular complex with a 1:1 molar ratio between ketones and *l*-menthol [76]. Green* et al.* observed emerging optically active poly(*n*-hexyl isocyanate) (PHIC) with a preferred handed helix in non-racemic chlorinated solvents [77]. Yashima* et al.* reported generating optically active *cis*-polyphenylacetylene conveying carboxyl groups due to hydrogen bonding interactions with chiral amines in dimethyl sulfoxide [36,37,78]. Achiral CD-silent zinc bis-porphyrin gave rise to CD-active 1:1 host-guest complexation with chiral amines and chiral alcohols [79,80]. Viscous liquid crystalline media are also able to serve as chiral influences to efficiently generate optically active helical *π*-conjugated polymers [35,81]. We demonstrate, for the first time, the production of enhanced CD-amplitude aggregates from an achiral polysilane bearing *n*-propoxyphenyl group when chiral alcohols, though very expensive, are used as solvents [38]. The polysilane adopts a CD-silent helical conformation with an equal probability of left- and right-helices in the alcoholic media that relies on a double-well potential energy surface like aromatic/aliphatic ketones and PHIC. A possible pathway for this ICD is assumed to be chiral OH/O interactions [38]. In recent years, inexpensive enantiopairs of volatile terpenes without chemical modification, including limonene, pinene, and carvone, have received increasing attention as candidates for inducing optical activity in polymers [39,41,42,43,44,45,46] and supramolecules [40,82,83,84,85]. 

**Figure 1:**
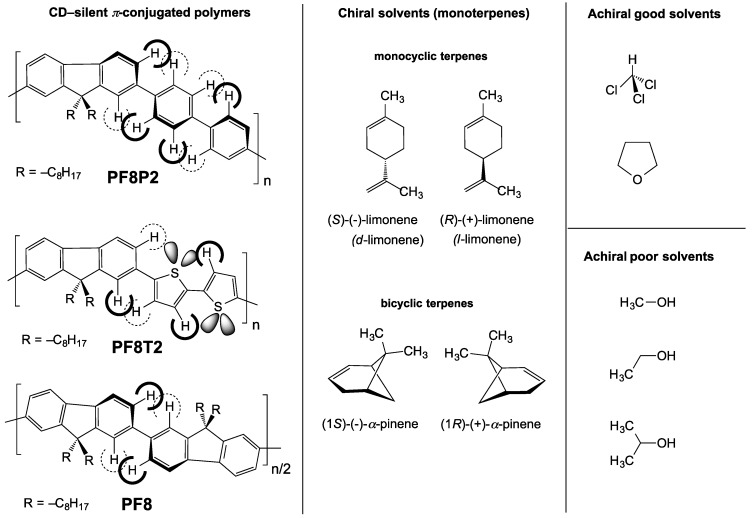
Chemical structure of three *π-*conjugated polymers (**PF8P2**, **PF8T2 [41]**, and **PF8** [42]) and chiral solvents (limonene and *α*-pinene) and good and poor achiral solvents.

Early limonene chirality transfer experiments were applied to well-designed host molecules, including carix[4]resorcarene [82] and dimeric porphyrin [83]. Alternatively, a vapor of chiral limonene and carvone, followed by a thermal annealing process, permits the induction of optical activity into syndiotactic polystyrene film, which is proven by ICD bands in the UV region and vibrational CD bands in the mid-IR region [39,84]. Limonene chirality transfer is possible to produce helical nanofiber and optically active supramolecular assemblies during gelation of perylene bisimide bearing achiral long alkoxybenzoylamide [85]. The uniqueness of our limonene solvent chirality transfer is the design of a cocktail, consisting of limonene and both poor and good achiral solvents, that allows for the successful emergence of several *π*-conjugated polymers (Figure 1) and *σ*-conjugated polymers as optically active polymer particles in a fluid solution with ≈100% recovery yield [38,41,42,43,44,45,46]. These aggregates provide exceptionally intense CD and/or circularly polarized luminescence (CPL) amplitudes in the UV-vis region, while the high photoluminescence (PL) quantum efficiency (*Φ*_PL_) of the aggregates is maintained close to that of the corresponding CD-/CPL-silent polymers dissolved in a homogeneous solution. Although these limonene chirality experimental results are reproducible, the scope and limitations along with a plausible explanation from a mechanistic viewpoint remain an open question.

To address these questions, here we focus on poly[(9,9-di-*n*-octylfluorenyl-2,7-diyl)-*alt*-biphenyl] (**PF8P2**), a simple soluble analog of poly-*p*-phenylene in the polyfluorene family [41,42,43,44]. The present paper reports the following MSB mechanisms: (i) through non-covalent intermolecular forces, the CD-/CPL-active **PF8P2** aggregate is instantly generated in a chiral tersolvent system consisting of chloroform (a good solvent), alkanols (poor solvents) and limonene (a chiral solvent); (ii) the chemical structure of alkanols and enantiopurity of limonene greatly affect the magnitude and sign of the CD-/CPL-signal amplitude; (iii) clockwise (CW) and counterclockwise (CCW) stirring during the preparation of aggregates considerably affects the CD magnitudes by a factor of two; (iv) the stirring speed greatly affects the CD amplitude and sign; and (v) the order of addition of limonene and methanol to the chloroform solution of **PF8P2** greatly affects the magnitude and sign of the CD amplitude.

## 2. Results and Discussion

In recent years, the generation of aggregation-induced CD, optical rotation (OR), optical rotation dispersion (ORD) and CPL signals through the help of chiral substituents, additives and solvents has generated much interest among molecular, materials, and polymer scientists [33,34,38,41,42,43,44,46,85,86]. These chiroptical enhancements originate from so-called exciton coupling [79,87] between the nearest neighbors due to dipole-allowed transitions, and, in some cases, dipole-forbidden transitions. 

### 2.1. UV-Vis, PL and CD Spectra of PF8P2 Homogeneously Dissolved in Dilute Solutions at Room Temperature

The UV-vis and PL spectral characteristics (*λ*_max_, molar absorptivity (*ε*), and *λ*_lum_) of **PF8P2** in chloroform are blue-shifted by 30 nm compared to those of poly[(9,9-di-*n*-octylfluorenyl-2,7-diyl) (**PF8**). Either **PF8P2** or **PF8** is in common *π-*conjugated aromatic polymers without any heteroatoms in the main-chain and side chains. **PF8P2** absorbs with *λ*_max_ at 363 nm and emits with *λ*_lum_ at 408 nm with a relatively large Stokes’ shift of 2920 cm^−1^, while **PF8** (1.0 × 10^−5^ mol L^−1^) has *λ*_max_ at 386 nm and *λ*_lum_ at 417 nm with a smaller Stokes’ shift of 1640 cm^−1^. The *λ*_max_ of **PF8P2** is greatly blue-shifted by 1570 cm^−1^ (23 nm) compared to that of **PF8**. These UV-vis and PL characteristics are inherently due to partial loss of the main-chain coplanarity of **PF8P2** compared to that of **PF8** (see Figure 1); the free-rotation ability along numbers of biphenyl ring axes is particularly crucial.

**Figure 2:**
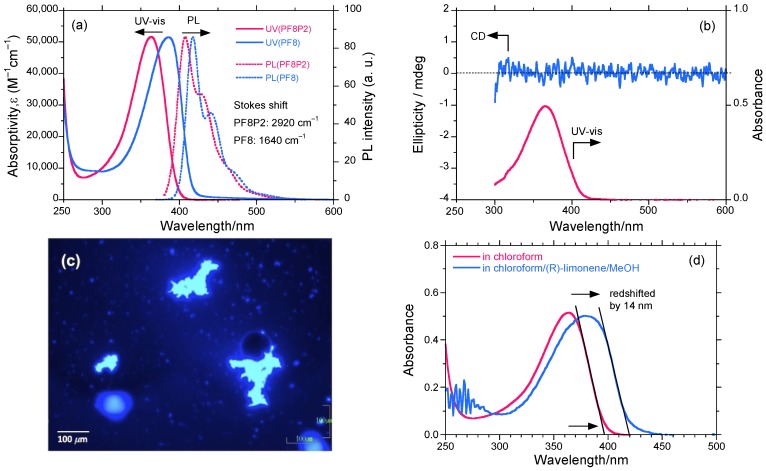
(**a**) The UV-vis absorption (1.0 × 10^−5^ mol L^−1^) and PL spectra (excited at 360 nm, 1.0 × 10^−7^ mol L^−1^) of **PF8P2** in chloroform (red solid and dotted lines), and for comparison, normalized UV-vis (ca. 2 × 10^−5^ mol L^−1^) and PL (excited at 360 nm, 1 × 10^−7^ mol L^−1^) spectra of **PF8** in chloroform (blue solid and dotted lines). (**b**) The CD and UV-vis spectra (1.0 × 10^−5^ mol L^−1^) of **PF8P2** in chloroform/(*R*)-limonene (0.3/2.7 (v/v)) at 25 °C. (**c**) PL image (excited at 365 nm) of **PF8P2** aggregates produced in chloroform/limonene/methanol (0.3/1.1/1.6 (v/v/v)). Scale bar: 100 *μ*m. (**d**) The red-shifted UV-vis spectra of **PF8P2** due to aggregation in chloroform/(*R*)-limonene/methanol (0.3/1.1/1.6 (v/v/v)).

Figure 2a shows the UV-vis (1.0 × 10^−5^ mol L^−1^) and PL spectra (excited at 360 nm, 1.0 × 10^−7^ mol L^−1^) of **PF8P2** in chloroform at 25 °C, and for comparison, the normalized UV-vis and PL spectra of **PF8** in chloroform. Figure 2b shows the UV-vis (1.0 × 10^−5^ mol L^−1^) and CD spectra (1.0 × 10^−5^ mol L^−1^) of **PF8P2** in chloroform/(*R*)-limonene (0.3/2.7 (v/v)) at 25 °C.

From Figure 2b, the *λ*_max_ of 366 nm of **PF8P2** in chloroform/(*R*)-limonene is weakly red-shifted by 3 nm due to the subtle effect of the less polar limonene. It is worth noting that neither **PF8P2** nor **PF8** in homogeneously dissolved chloroform exhibits detectable Cotton CD effects in the region of* π*-π* transitions due to the lack of efficient chiral chemical influence regardless of the 90% (*R*)-limonene in the (*R*)-limonene/chloroform cosolvent. These spectroscopic properties feature a free-rotation ability between two phenyl rings of a biphenyl linkage and between the fluorene and biphenyl rings of **PF8P2** (see Figure 1), leading to a partial loss of *π*-conjugation regardless of the presence of a significant amount of limonene.

Figure 2c shows the fluorescence optical microscopy image of **PF8P2** aggregates made by chloroform/(*R*)-limonene/methanol [0.3/1.1/1.6 (v/v/v)] under CW 800 rpm stirring. The aggregate size is typically 10–50 μm. For the **PF8P2** aggregates in an aggregation process using chiral tersolvents, these dynamic twisting abilities lead to a great susceptibility to subtle differences in the chemical structures of the chiral and achiral solvents. Even the mechanical stirring conditions with a magnetic bar within a quartz cuvette including stir direction and stir speed can have an effect, as shown in later sections.

Figure 2d shows the UV-vis spectroscopic evidence of *J-*aggregation [41,42,43,44,87,88] and extended coplanarity of **PF8P2** during aggregation using chloroform/limonene/methanol [0.3/1.1/1.6 (v/v/v)]. Evidently, the apparent *π*-π* absorption edge is red-shifted by 1380 cm^−1^ (14 nm) due to aggregation. The *J-*type, extended coplanar *π*-conjugation of the **PF8P2** main-chain might be responsible for the relatively high *Φ* values even in aggregated structures, as demonstrated in later sections.

### 2.2. UV-Vis, Photoluminescence (PL) and CD Spectra of PF8P2 Aggregates in Fluidic Solvents

**Figure 3:**
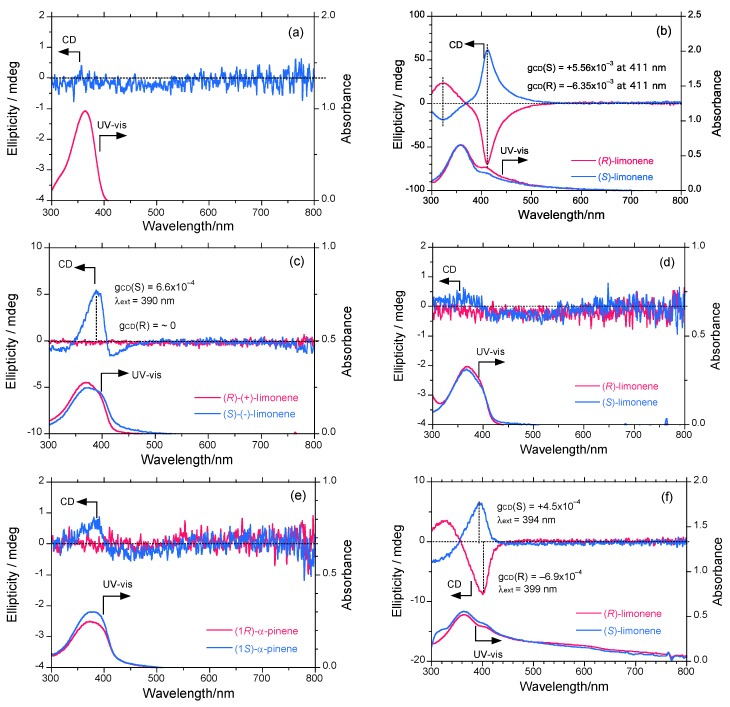
The CD and UV-vis spectra of **PF8P2** in (**a**) chloroform/methanol = 0.3/2.7 (v/v), (**b**) chloroform/(*R*)- and (*S*)-limonene/methanol = 0.3/1.1/1.6 (v/v/v), (**c**) chloroform/ (*R*)- and (*S*)- limonene/ethanol = 0.3/1.1/1.6 (v/v/v), (**d**) chloroform/(*R*)- and (*S*)-limonene/ isopropanol = 0.3/1.1/1.6 (v/v/v), (**e**) chloroform/(1*R*)- and (1*S*)-*α*-pinene/methanol = 0.3/1.1/1.6 (v/v/v), (**f**) tetrahydrofuran/(*R*)- and (*S*)-limonene/methanol = 0.3/1.1/1.6 (v/v/v). [**PF8P2**]_0_ = 1.0 × 10^−5^ mol L^−1^ and measurements are employed at 25 °C. Stirring conditions: CW stirring operation at 800 rpm.

Figure 3a shows the CD and UV-vis spectra (1.0 × 10^−5^ mol L^−1^) of the **PF8P2** aggregate generated in chloroform/methanol = 0.3/2.7 (v/v) at 25 °C under CW stirring at 800 rpm. The **PF8P2** aggregate *λ*_max_ at 369 nm is red-shifted by 6 nm compared to that in isotropic chloroform solution, though there are no detectable CD signals due to the absence of chiral chemical influence. The red shift is assumed to originate from the *J*-type aggregation of chromophores that enable the maintenance of highly PL properties. This effect might be caused by the inhibition of non-emissive face-to-face *π*-*π* stacks and emissive slipped *π*-*π* stacks due to the intramolecular CH/*π* interaction of 9,9-dialkylfluorene derivatives reported recently [89].

Figure 3b shows the CD and UV-vis spectra (1.0 × 10^−5^ mol L^−1^) of the **PF8P2** aggregate generated in chloroform/limonene/methanol = 0.3/1.1/1.6 (v/v/v) at 25 °C under the 800 rpm-CW stirring operation. The relative fraction of tersolvents is optimized prior to a series of terpene chirality transfer experiments, as discussed in a later section. The **PF8P2** aggregate exhibits intense apparent bisignate-type Cotton CD signals (*λ*_ext_: ≈ 411 and ≈ 320 nm), and these bands cross over at 368 nm. The 411-nm CD band is consistent with the UV-vis band* λ*_max_ at 411 nm, but the 320-nm CD band is not consistent with the corresponding UV band. Limonene does not show chiroptical inversion effects [45]. The 411-nm CD band originates from main-chain parallel dipole-allowed *π*-π* transition moments, while the 320-nm CD band may arise from main-chain perpendicular dipole-forbidden *π*-π* transition moments.

The **PF8P2** aggregate has a *λ*_max_ at 411 nm, which is greatly red-shifted by 2770 cm^−1^ (42 nm) compared to that in the limonene-free aggregates in the chloroform-methanol cosolvents. The red-shift is thought to be a consequence of increasing the effective *π*-conjugation by generating coplanarity between two biphenyl groups and between the biphenyl and fluorene rings. Another possibility is that it originates from the *J*-aggregation of chromophores that are capable of keeping the original highly PL property. This coplanarity may be associated with the intermolecular stacking ability between multiple main-chains with the help of intermolecular non-covalent weak forces between **PF8P2**, the poor solvent methanol and chiral limonene molecules. Non-covalent weak interactions are assumed to be *π*/*π*, van der Waals, and CH/*π* interactions [89,90,91,92], though experimental proof for these has yet to be elucidated. Recently, we reported the existence of an intramolecular CH/*π* interaction between *β*-CH_2_ protons at the 9,9-position of the fluorene *π*-ring in several 9,9-dialkylfluorene derivatives by means of two-dimensional ^1^H-NMR NOESY spectra and second-order Møller-Plesett (MP2) perturbation calculations [Gaussian03, 6-311G(d) basis set] [89]. Non-covalent inter- and intramolecular CH/*π* and *π-π* interactions are assumed to be responsible for the high PL property [41,42,43,44], regardless of the presence or absence of limonene, discussed later. The non-covalent weak forces including the intramolecular CH/*π* interaction should allow the inhibition of non-emissive face-to-face *π-π* stacks. These three sets of interactions should provide MSB PL polymer aggregates in fluidic media.

A mechanistic insight of limonene-driven chiral aggregation from **PF8P2** is at the present stage speculative. In a series of the limonene induced polymer aggregation experiments and theoretical calculations [33,41,42,43,44], we assume that multiple H–H repulsions between biphenyl-like structures within main-chains of **PF8P2**, **PF8T2** [41], **PF8** [42], and their analogs [33,43,44] are responsible for twisting ability with handedness that is susceptible to solvent chirality during aggregation process. The H–H repulsions commonly exist **PF8P2**, **PF8T2**, **PF8**, and their analogs [33,41,42,43,44]. The intramolecular CH/*π* interaction between *β*-CH_2_ protons of two *n*-octyl chains at the 9,9-position and the fluorene *π*-ring in these 9,9-dialkylfluorene derivatives may help an induction of the corresponding optical active, *J*-type slipped stacks due to self-wrapping effect [89]. In addition, the length of *n*-alkyl group of 9,9-dialkylfluorene derivatives is important. Actually, our previous limonene chirality transfer experiments using a series of poly(9,9-di-*n-*alkylfluorene)s (alkyl; *n*-hexyl, *n*-heptyl, *n*-octyl, *n*-decyl, *n*-dodecyl, *n*-hexadecyl, *n*-octadecyl), **PF8** and poly(9,9-di-*n-*decylfluorene) in these aggregates provided exceptionally intense CD amplitude signals in π-π* main-chain absorption bands, indicating that limonene chirality are efficiently transferred to the aggregates with the help of multiple van der Waals, CH-*π*, and *π*-*π* interactions [42]. A possible reason for this *n-*alkyl chain length dependency is that molecular length of limonene long axis is almost identical to that of *n*-octyl and *n*-decyl groups, thereby, leading to efficient van der Waals, CH-*π*, and *π*-*π* interactions between the alkyl groups and limonene molecules [42]. The choice of proper *n-*alkyl chain length is crucial for generating optically active polymer aggregates with further help of a poor solvent.

Figure 3c shows the CD and UV-vis spectra (1.0 × 10^−5^ mol L^−1^) of the **PF8P2** aggregate generated in chloroform/limonene/ethanol = 0.3/1.1/1.6 (v/v/v) under the same conditions. Much to our surprise, (*S*)-limonene induces a weak, detectable CD signal with a *g*_CD_ value of +6.6 × 10^−4^ at 390 nm within the **PF8P2** aggregate, while (*R*)-limonene does not induce such a signal. The *λ*_max_ values of (*S*)- and (*R*)-limonene are slightly different from each other. For the (*S*), 372 nm is the peak and 397 nm is the shoulder, while for the (*R*), 368 nm is the only peak. 

Figure 3d shows the CD and UV-vis spectra (1.0 × 10^−5^ mol L^−1^) of the **PF8P2** aggregate generated in chloroform/limonene/isopropanol = 0.3/1.1/1.6 (v/v/v) under the same conditions. (*S*)-limonene may induce a weak detectable CD signal, but (*R*)-limonene does not. Figure 3e shows the CD and UV-vis (1.0 × 10^−5^ mol L^−1^) of the **PF8P2** aggregate generated in chloroform/*α*-pinene/methanol = 0.3/1.1/1.6 (v/v/v) under the same conditions. To our surprise, only (1*S*)-pinene induces a weak detectable CD signal; (1*R*)-pinene does not.

Finally, Figure 3f shows the CD and UV-vis spectra (1.0 × 10^−5^ mol L^−1^) of the **PF8P2** aggregate generated in tetrahydrofuran (THF)/limonene/methanol = 0.3/1.1/1.6 (v/v/v) under the same conditions. Evidently, (*S*)-limonene weakly induces a positive-sign CD signal showing a *g*_CD_ value of +4.5 × 10^−4^ at 394 nm, while (*R*)-limonene weakly induces a negative-sign CD signal showing a *g*_CD_ value of –6.9 × 10^−4^ at 399 nm, which is red-shifted by 5 nm.

A family of tersolvent cocktails, including chloroform/limonene/ethanol, chloroform/limonene/ isopropanol, chloroform/*α-*pinene/methanol, and THF/limonene/methanol thus induces local MSB effects within a cuvette due to unknown reasons. There is a possibility to generate a preferred optically active structure between two possible mirror-image polymers. Similar local MSB effects for several optically active molecular crystals, oligomers, and polymer aggregates have been reported recently, when an equality in physical properties between an (*S*)-(*R*) pair is compared [44,61,62,66,68,69,71]. 

A possible explanation of this phenomenon arises from certain chiral chemical impurities in the terpenes. However, (*S*)- and (*R*)-limonenes obtained by reduced distillation are analytically pure by our chiral gas chromatography and optical rotation measurements [41,42] (see Experimental section). The other possible origin is *Z*°-boson induced parity-violating weak nuclear force within chiral and achiral substances (**PF8P2**, terpenes, and alcohols), in which this force is classified as the global MSB effect due to inherent handedness, though it is thought to be extremely weak in theory and on the order of 10^−^^8^–10^−^^14^*J* mol^−^^1^ [50,51,52,53,54,55,56,57,58,59,63,64,65,59,63]. Although the source of these anomalies might be a source of *much* debate, an elucidation of the origin remains an issue for future research.

### 2.3. Search for Optimized Conditions to Generate Optically Active PF8P2 Aggregates

**Figure 4:**
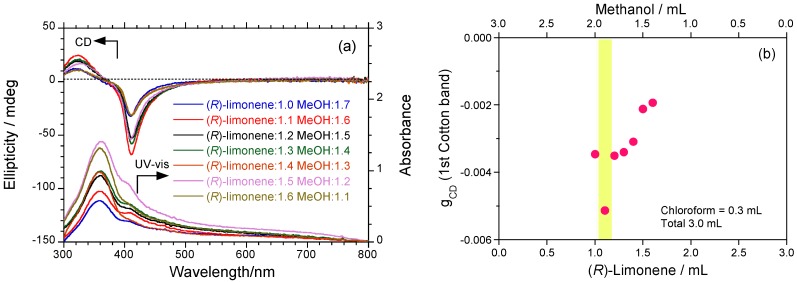
(**a**) Changes in the CD and UV-vis absorption spectra of **PF8P2** aggregates in chloroform (0.3 mL fixed) simultaneously varying the volume fractions of (*R*)-limonene and methanol. (**b**) The *g*_CD_ value at ~405 nm as a function of volume fractions of (*R*)-limonene and methanol. [**PF8P2**] = 1.0 × 10^−5^ mol L^−1^.

As reported in a series of our reported research, optically active **PF8P2** aggregates generated by terpene chirality transfer are susceptible to a relative volume fraction of (*R*)-limonene and methanol, when the chloroform and total volume of the mixed solvent are fixed at 0.3 mL and 3.0 mL, respectively. Figure 4a shows the change in the CD and UV-vis spectra of optically active **PF8P2** aggregates when varying a relative volume fraction of (*R*)-limonene and methanol. Figure 4b plots the *g*_CD_ at the first Cotton CD band as a function of methanol and (*R*)-limonene. The absolute magnitude of the *g*_CD_ is maximized at very specific volume fractions of (*R*)-limonene and methanol. The reason for this specificity is ascribed to the ‘chiral optofluidic effect’ recently reported [44]. The chiral optofluidic effect denotes fine tunings in refractive indices (RIs) between polymer particles with a higher RI and the surrounding fluidic media with a lower RI and in optical rotation between optically active polymer particles and the surrounding optically active fluidic media. This very specific tersolvent fraction was applied to other aggregation experiments including (*S*)-limonene and (1*S*)-/(1*R*)-*α*-pinenes.

### 2.4. UV-Vis, PL, CD, and CPL Spectra of PF8P2 Aggregates Generated under Optimized Conditions

**Figure 5:**
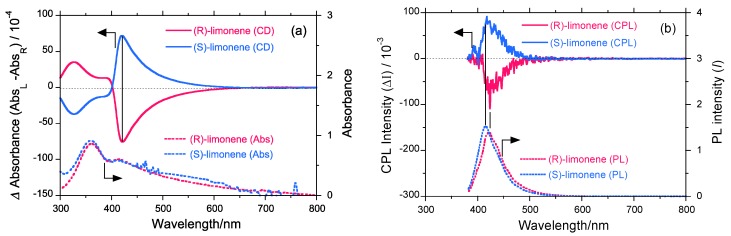
(**a**) The raw CD and UV-vis (1.0 × 10^−5^ mol L^−1^) and **(b)** CPL and PL spectra of **PF8P2** aggregates generated under optimized chloroform/limonene/methanol (=0.3/1.1/1.6 (v/v/v)) conditions at 25 °C.

Figure 5a shows the CD and UV-vis spectra (1.0 × 10^−5^ mol L^−1^) of **PF8P2** aggregates generated under the optimized chloroform/limonene/methanol (0.3/1.1/1.6 (v/v/v)) conditions. **PF8P2** aggregates reveal the greatest *g*_CD_ values in several experiments. In (*S*)-limonene, the *g*_CD_ = +1.22 × 10^−2^ is at 419 nm and in (*R*)-limonene, the *g*_CD_ = −1.25 × 10^−2^ is at 421 nm. Figure 5(b) displays the CPL and PL spectra of **PF8P2** aggregates generated under the optimized conditions. **PF8P2** aggregates reveal the greatest *g*_CPL_ values in (*S*)-limonene, *g*_CD_ = +5.7 × 10^−3^ at 415 nm and in (*R*)-limonene, *g*_CD_ = –5.8 × 10^−3^ at 423 nm. The absolute magnitudes of *g*_CPL_ are almost half those of the *g*_CD_ values. CPL and CD denote chirality in the excited and the ground states, respectively. The excited chirality may partly lose its chirality by reorganizing chirally assorted structures and/or racemization of twisted C-C bonds between biphenyl and fluorene rings. 

Nevertheless, it is noteworthy that, regardless of the lack of stereocenters in the main- and side-chains of **PF8P2**, limonene chirality is able to induce optical activity with distinct amplitudes into the polymer aggregates as a suspension forms in fluidic media at 25 °C within ≈10 sec without any loss of polymer samples. This occurs because an aggregation process is inherently a loss-less technique to recover all polymer dissolved in a good solvent by adding a poor solvent to re-precipitate. This is definitively an environmentally friendly, safer, and milder process to instantly produce ambidextrous light-emitting *π-*conjugating polymers with minimal loss from the corresponding optically inactive *π-*conjugating polymers used as starting material at ambient temperature without any chiral substituents and/or chiral catalysts, although naturally occurring chiral terpenes at solvent quantity are needed. The chiral terpenes used are renewable by reduced distillation.

### 2.5. Limonene Homochirality Affects PF8P2 Aggregation

In terms of biomolecular handedness in nature, non-linear chiroptical amplification within isolated polymer chains and *π-π* supramolecular stacks, which is well-known as being the predominant type, is fascinating. This is called the “positive nonlinear cooperativity effect” [93]. Optically active polymers and supramolecular *π-π* stacks with a preferred helix sense are nonlinearly amplified with a positive sense as a function of the *ee* value of chiral pendant groups [94]. On the other hand, we showed that, in the **PF8T2-**limonene system, almost 100% enantiopure limonene is inevitably needed to efficiently generate optically active **PF8T2** aggregates [41]. The homochiral nucleation and growth with the same limonene chirality is preferable to the hetereochiral nucleation and growth with the antipode chirality of limonene. This is due to the “negative nonlinear cooperativity effect” [93].

**Figure 6:**
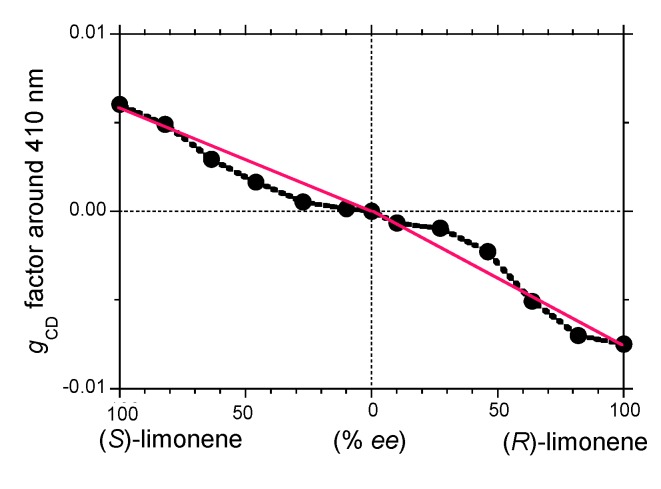
The *g*_CD_ value at the 405 nm CD band of **PF8P2** aggregates in chloroform/limonene/ methanol = 0.3/1.1/1.6 (v/v/v) as a function of limonene % *ee*.

Figure 6 plots the *g*_CD_ value as a function of the *ee* value of limonene generated under the optimized conditions with 800 rpm CW stirring operation. Evidently, the *g*_CD_ value increases inefficiently with an increase of the *ee* value of limonene. A negative nonlinear cooperativity of **PF8P2** can be seen as well as **PF8T2**. The curve of the *g*_CD_ value in the (*S*)-limonene-rich region, however, is somewhat different from that in the (*R*)-limonene-rich region. A comparison of the (*S*)-limonene-*g*_CD_ and (*S*)-limonene-*g*_CD_ curves implies the occurrence of a weak MSB effect.

### 2.6. Vortex Stirring Direction and Speed Affect PF8P2 Aggregation

Early vortex stirring experiments reported the emergence of optically active supramolecular aggregates of water-soluble porphyrin derivatives and a certain Rhodamine B doped thermo-responsive polymer with sol-gel transition ability [21,22,23,24,25]. Little effect of the vortex stirring direction and/or stirring speed dependency in fluidic media has been reported to date. 

**Figure 7:**
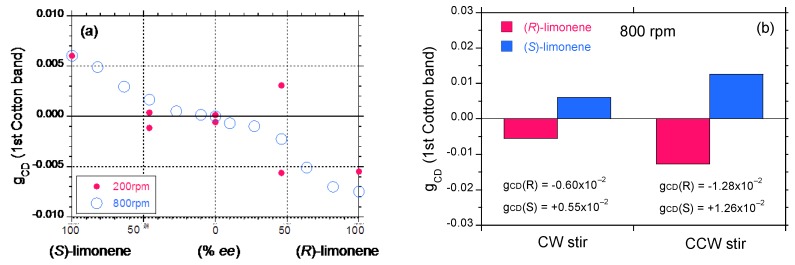
(**a**) Stirring speed (200 and 800 rpm) dependency of the *g*_CD_ value at the 405 nm CD band of **PF8P2** aggregates as a function of limonene % *ee* in chloroform/(limonene+methanol) = 0.3/2.7 (v/v) under CW stirring. Open blue and red filled circles are 800 rpm and 200 rpm, respectively. (**b**) Stirring direction (CW and CCW) dependency (800 rpm) of the *g*_CD_ value at the 405 nm CD band of **PF8P2** aggregates in chloroform/(*S*)- and (*R*)-limonene/methanol = 0.3/1.1/1.6 (v/v/v). Red and blue bars are (*R*)-limonene and (*S*)-limonene, respectively.

Figure 7a plots the *g*_CD_ value as a function of the *ee* value of limonene in chloroform/(*R*)- and (*S*)-limonene/methanol = 0.3/1.1/1.6 (v/v/v) under CW stirring operation at 200 and 800 rpm. Under slow 200 rpm stirring, the *g*_CD_ values in magnitude and sign fluctuated largely with the limonene *ee* value. Particularly, at 50% *ee* of (*R*)-limonene, the *g*_CD_ values were widely scattered in the positive and negative region. This feature might result from stochastic resonance in a bimodal left-or-right distribution as a matter of chance [10,11,13,15,16,17]. An initial subtle left-right fluctuation at the bifurcation point in a double-well potential definitively determines the subsequent left-right selection. This is an absolutely random process macroscopically. On the other hand, under vigorous 800 rpm stirring, the *g*_CD_-*ee* curves in both the (*S*)- and (*R*)-limonene regions is nearly symmetric, though the weak MSB effect between (*R*)- and (*S*)-limonene regions can be seen.

However, much to our surprise, the *g*_CD_ values of **PF8P2** aggregates generated with the 800 rpm CCW stirring operation are almost twice those generated with the 800 rpm CW stirring operation, which is independent of limonene chirality due to unknown reasons. The absolute *g*_CD_ value under the 800 rpm CW stirring is typically ≈0.6 × 10^−^^2^, while the value is ≈1.3 × 10^−^^2^ under the 800 rpm CCW. Although the majority of our work employed the 800 rpm CW stirring operation, the CCW stirring operation might be an alternative option to obtain more intense optically active polymer aggregates in the future.

A very speculative explanation of this anomaly that **PF8P2** and limonene molecule may preferentially rotate in the left- or right-handed direction along their multiple single bond axes. External mechanophysical twisting bias may act as an additive or a subtractive force to the preferential rotating direction at the polymer and molecular levels. Although the source of these anomalies might be a source of *much* debate, an elucidation of the origin remains an issue for future research.

### 2.7. Addition Order Dependency of Limonene and Methanol Affecting PF8P2 Aggregation

To test the chiroptical stability of optically active **PF8P2** aggregates, we employed two addition modes of limonene chirality in a similar fashion to **PF8T2** [41]. To the (*R*)-limonene-induced **PF8P2** aggregates, (*S*)-limonene was then added; conversely, to the (*S*)-limonene-induced **PF8P2** aggregates, (*R*)-limonene was added.

For the (*S*)-to-(*R*)-limonene experiment, 0.55 mL of (*R*)-limonene was added to 0.3 mL of a chloroform solution containing **PF8P2** in the cuvette. The addition of 1.6 mL of methanol resulted in the formation of optically active **PF8P2** aggregates. This volume fraction of chloroform/ (*R*)-limonene/methanol [0.3/0.55/1.6 (v/v/v)] is not the optimized tersolvent used to generate intense optically active **PF8P2** aggregates. The insufficient (*R*)-limonene quantity, leading to loosely stacked π-π aggregates, allows the addition of (*S*)-limonene as a solvent. To the solution of **PF8P2** aggregates, 0.55 mL of (*S*)-limonene was added. This protocol was applied to the (*R*)-to-(*S*)-limonene **PF8P2** aggregates. In both cases, the final volume fraction of chloroform/limonenes/methanol was kept at 0.3/1.1/1.6 (v/v/v), in which these limonenes are macroscopically in racemic. 

**Figure 8:**
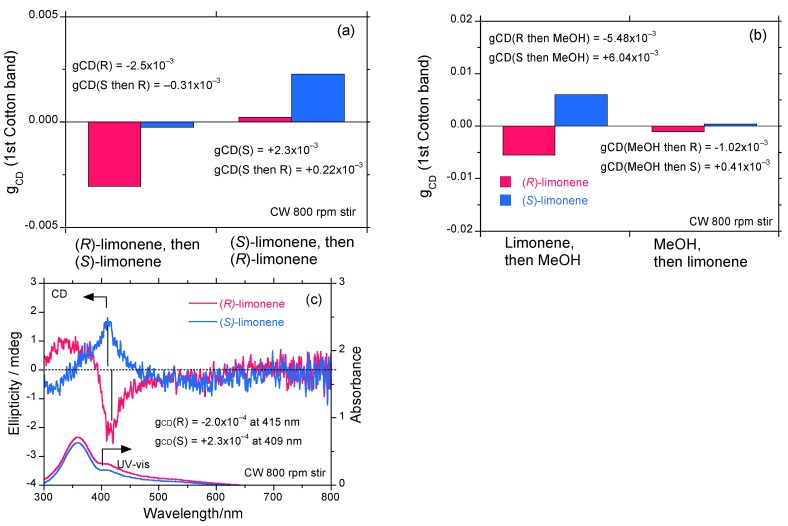
(**a**) The (*S*) and (*R*) limonene addition order dependency of the *g*_CD_ value of **PF8P2** aggregates in chloroform/limonene/methanol = 0.3/1.1/1.6 (v/v/v). The two left bars depict the (*R*)-limonene addition to (*S*)-limonene-induced **PF8P2** aggregates; the two right bars are the (*S*)-limonene addition to (*R*)-limonene-induced **PF8P2** aggregates. (**b**) The methanol and limonene addition order dependency of the *g*_CD_ value of **PF8P2** aggregates in chloroform/limonene/methanol = 0.3/1.1/1.6 (v/v/v). The two left bars depict methanol addition to limonene-induced optically active **PF8P2** aggregates; the two right bars are the limonene addition to methanol-induced optically inactive **PF8P2** aggregates. Red and blue bars are (*R*)- and (*S*)-limonene, respectively. **(c)** The CD and UV-vis spectra of optically active **PF8P2** aggregates generated by the limonene-to-methanol addition.

Figure 8a shows a comparison of the *g*_CD_ values of the (*S*)-to-(*R*) and (*R*)-to-(*S*) limonene experiments. For the (*S*)-to-(*R*) experiments, the *g*_CD_ value of −2.5 × 10^−3^ diminishes eight-fold while maintaining a negative sign. In contrast, in the (*R*)-to-(*S*) experiments, the *g*_CD_ value of +2.3 × 10^−3^ greatly diminishes by ten-fold while maintaining a positive sign. By contacting the opposite limonene chirality in fluidic conditions, optically active **PF8P2** aggregates easily lose their optical activity.

Another idea is to test which process induces optical activity into **PF8P2** aggregates in a similar manner to **PF8T2** [41]. To do this, we employed two addition modes between limonene and methanol to a chloroform solution of **PF8P2** to generate optically active polymer aggregates. The normal addition method used in this work is the slow addition of methanol to a homogeneous chloroform/limonene solution containing **PF8P2** to produce aggregates (see the Experimental section). The reverse addition is the addition of limonene to the inhomogeneous chloroform–methanol medium containing **PF8P2** aggregates, in which optically inactive or CD-silent aggregates are formed as a suspension in fluidic media. The final volume fraction of chloroform/limonene/methanol is kept at 0.3/1.1/1.6 (v/v/v).

The reverse addition mode produces a similar bisignate CD/UV-vis spectral profile to the normal addition mode. As shown in Figure 8b, the *g*_CD_ values by the reverse mode is reduced by 5–10 times compared to those obtained by the normal mode, regardless of limonene chirality. The limonene chirality appears to be sensitive to the addition order. These experiments indicate that the nucleation and initial aggregation process due to limonene chirality is definitively important during the addition of a poor solvent, methanol.

Figure 8c shows a comparison of the CD and UV-vis spectra between the optically active **PF8P2** aggregates with (*S*)- and (*R*)-limonene chirality by the reverse addition mode under 800 rpm CW stirring operation. The Cotton CD spectral profiles of (*S*)- and (*R*)-limonenes are very different from each other. (*R*)-Limonene induces the **PF8P2** aggregates with a longer *λ*_ext_ of 415 nm, which is red-shifted by ≈6 nm compared to that of (*S*)-limonene induced **PF8P2** aggregates. A weak MSB effect may take place.

### 2.8. Photodynamics of Limonene-Induced PF8P2 Aggregation

As displayed in Figure 2c, the size of **PF8P2** aggregates was typically 10–100 *μ*m. However, considerable differences in PL spectra, *Φ*_ΠΛ_ value, and PL decay curves between: (i) molecules dissolved in chloroform, (ii) aggregates suspended in chloroform/(*R*)-limonene/methanol (0.3/1.1/1.6 (v/v/v)) and (iii) aggregates suspended in chloroform/(*S*)-limonene/methanol can be seen. **PF8P2** in chloroform has a considerably high *Φ*_ΠΛ_ of 32%. **PF8P2** aggregates in chloroform/(*R*)-limonene/ methanol and chloroform/(*S*)-limonene/methanol are 20% and 16%, respectively, compared to the reference anthracene (*Φ*_ΠΛ_ = 30% in ethanol). Thus, **PF8P2** has high *Φ*_ΠΛ_ values regardless of isolated polymer and aggregate states. This feature could be a consequence of steric demanding effects due to intramolecular CH/*π* interaction of 9,9-dialkylfluorene rings [89]. 

**Figure 9:**
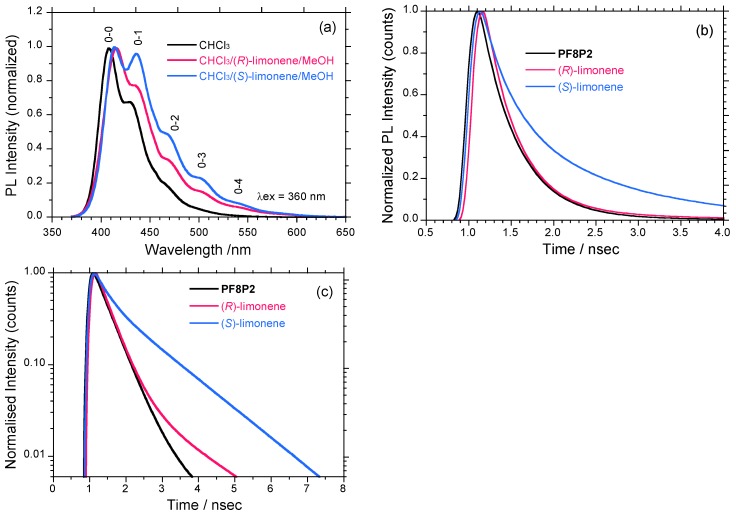
(**a**) Normalized PL spectra excited at 360 nm of **PF8P2** (1 × 10^−7^ M) in chloroform (black line), **PF8P2** aggregates in chloroform/(*R*)-limonene/methanol (0.3/1.1/1.6 (v/v/v), red line) and **PF8P2** aggregates in chloroform/(*S*)-limonene/methanol (0.3/1.1/1.6 (v/v/v), blue line). (**b**) and (**c**) PL decay curves (excited at 360 nm) collected at 380-420 nm of **PF8P2** and aggregates as a function of time. The ordinates are plotted as (b) linear and (c) log scales.

Figure 9a compares the following three PL spectra (excited at 360 nm): **PF8P2** in chloroform, **PF8P2** aggregates in the chloroform/(*R*)-limonene/methanol, and the chloroform/(*S*)-limonene/ methanol. The *λ*_lum_ of the aggregates is slightly red-shifted by 415 cm^−1^ (8 nm). However, a significant difference between the three PL spectra is that **PF8P2** aggregates in the chloroform/(*S*)-limonene/ methanol have four clearer vibronic phonon side bands (0–1, 0–2, 0–3, 0–4) compared to other two **PF8P2** systems. Electron-phonon coupling with the aggregates in the chloroform/(*S*)-limonene/ methanol is much stronger than that of other two systems.

Figure 9b,c display three PL decay curves collected at 380–420 nm of **PF8P2** and its aggregates as a function of time. The ordinates are plotted on linear and log scales. From PL decay analysis, **PF8P2** in chloroform rapidly decays almost exponentially but through two decay channels with lifetimes of 0.435 nsec (0.179) and 1.832 nsec (0.0027). The major PL component is the 0.435 nsec decay channel. Similarly, **PF8P2** aggregates in the (*R*)-limonene tersolvent also rapidly decay with two decay channels with lifetimes of 0.388 nsec (0.152) and 1.637 nsec (0.0073). The major PL component is the 0.388 nsec decay channel. However, **PF8P2** aggregates in the (*S*)-limonene tersolvent decay non-exponentially with two lifetimes of 0.342 nsec (0.091) and 1.401 nsec (0.072). The **PF8P2**-(*S*)-limonene system has two major shorter and longer PL components with an almost equal contribution. Thus, PL decay characteristics of **PF8P2** aggregates generated in the (*S*)- and (*R*)-limonene tersolvents are no longer in a mirror-image relationship. An MSB effect on PL decay characteristics in the (*S*)-(*R*) limonene induced **PF8P2** aggregates may take place, though the origin is still unknown.

## 3. Experimental

### 3.1. Synthesis of PF8P2

**Scheme 1:**
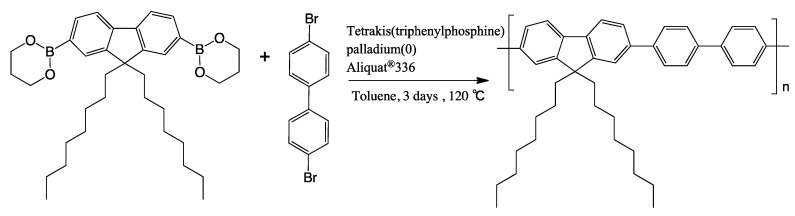
Synthesis route of poly[(9,9-dioctylfluorenyl-2,7-dinyl)-*alt*-biphenyl] (**PF8P2**).

The synthesis of **PF8P2** has already been reported by Ranger and Leclerc [95]. A reaction scheme is given in Scheme 1. 

**Figure 10:**
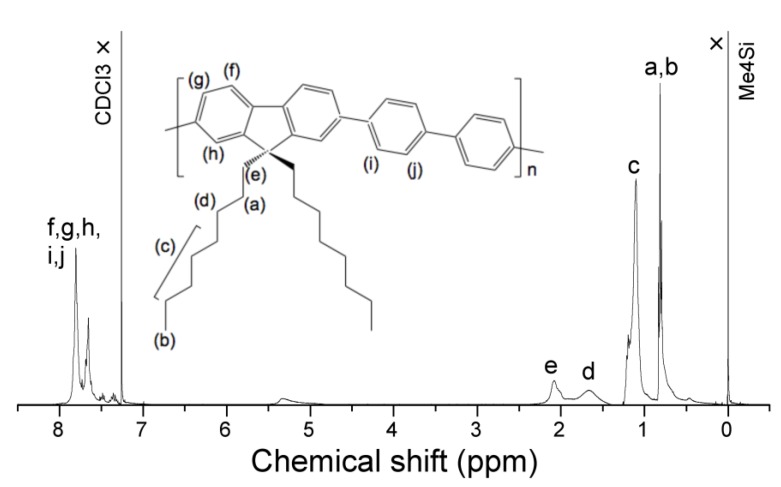
^1^H-NMR spectrum of **PF8P2** in CDCl_3_ at 25 °C, (CH_3_)_4_Si as an internal standard (399.00 MHz).

To a 100-mL three-necked flask, dioctylfluorene-2,7-diboronic acid (Sigma-Aldrich, St. Louis, MO, USA), 0.855 g, 1.53 mmol), 4,4'-dibromobiphenyl (Tokyo Chemical Industry (TCI), Tokyo, Japan), 0.479 g, 1.53 mmol), tetrakis(triphenylphosphine)palladium(0) (Sigma-Aldrich, 0.18 g, 0.015 mmol) were added. After purging with pure N_2_ gas, Aliquat^®^336 (Sigma-Aldrich, 0.07 mL, 0.15 mmol) and dehydrated toluene (Wako Chemical (Wako), Osaka, Japan), 10 mL) were added. The mixture was allowed to react at 120 °C with gentle stirring with a magnetic stir bar, followed by the addition of Na_2_CO_3_ (2 mol L^−1^) 3.60 mL and reacts for three days. To the reaction mixture, bromobenzene (TCI, 0.16 mL, 1.5 mmol) was added to terminate polymerization and further allowed to react for 12 h. A crude purple-white solid was isolated. Re-precipitation with methanol, twice, and filtration with 5/10 *μ*m-pore membrane filters provided a white solid. Isolated yield, 0.902 g (67.6%). Gel permeation chromatography analysis of **PF8P2** showed *M*_n_ = 1.1 × 10^4^, *M*_w_ = 3.3 × 10^4^, *M*_w_/*M*_n_ = 2.93. The *M*_n_ and *M*_w_ values, respectively, corresponded to *ca.* 20 and 65 repeating units by reference to polystyrene standards. As shown in Figure 10 and Table 1, the ^1^H-NMR spectrum and analysis fully agreed with the named polymer.

**Table 1:** Chemical shifts of **PF8P2** in CDCl_3_.

Chemical shift/ppm		Obs (H signals)	Calc (Assignment)
0.5–0.9	*t* + *br*	10.5	10H (*β*-CH_2_^[1]^ + *ω*-CH_3_)
1.1	*m*	17.6	16H (CH_2_)
1.6	*br*, *s*	3.3	16H (*γ*-CH_2_)
2.1	*br*, *s*	3.6	4H (*α*-CH_2_)
7.2-7.6	*m*	13.0	14H (Aromatic CH)

^[1]^ The unusual upfield shift of *β*-CH_2_ originates from* π*-ring current effect due to intramolecular CH/*π* interaction between fluorene ring and *β*-CH_2_ of *n*-octyl group [89].

### 3.2. Measurements

The CD/UV-vis spectra of the solutions were recorded simultaneously at 25 °C on a JASCO (Hachioji, Tokyo, Japan) J-725 spectropolarimeter with a Peltier-controlled unit using a synthetic quartz cuvette with an optical pathlength of 1.0 cm (scanning rate: 100 nm/min, bandwidth: 1 nm, response time: 1 sec, a single accumulation). UV-vis spectra were measured independently on a JASCO UV-550 UV-vis spectrophotometer at 25 °C (scanning rate: 100 nm/min, bandwidth: 2 nm, response time: 1 s). PL spectra were measured on a JASCO FP-6500 spectrofluorometer at 25 °C (scanning rate: 100 nm/min, excitation bandwidth: 3 nm, monitor bandwidth: 3 nm, response time: 1 sec, photomultiplier sensitivity: medium). The PL quantum yield was determined relative to anthracene (*Φ* = 30%, in ethanol). The CPL spectrum was recorded on a JASCO CPL-200 spectrofluoropolarimeter, with a path length of 10 mm at room temperature, while the instrument was designed to obtain a high S/N ratio by adjusting the angle between the incident and travelling light to 0° with a notch filter (scanning rate: 100 nm/min, slit width for excitation: 3000 μm, slit width for monitoring: 3000 *μ*m, response time: 1 s). Optical rotation at the Na-d line was measured with a JASCO P-1020 polarimeter with a path length of 1.0 cm at room temperature. The ^1^H NMR spectra was recorded with a JEOL (Akishima, Tokyo, Japan) EX-400 spectrometer at 400 MHz in CDCl_3_ at ≈24 °C. The weight-average molecular weight (*M*_w_), number-average molecular weight (*M*_n_) and polydispersity index (*PDI* = *M*_w_/*M*_n_) were evaluated using gel permeation chromatography (GPC) on a Shimadzu (Kyoto, Japan) A10 instrument with PLgel (Varian) 10 *μ*m mixed-B as the column and HPLC-grade THF as the eluent at 40°C (calibrated with polystyrene standards). The enantiopurity of limonene was determined using chiral gas chromatography, *β*-DEX-120, 30 mm × 0.25 mm ID (Supelco, St. Louis, MO, US), column oven temperature of 85 °C, He as a carrier gas with a flow rate of 1.2 mL min^−1^). Fluorescent optical micrographs excited at 365 nm were taken with a Nikon (Tokyo, Japan) Eclipse E400 optical microscope equipped with a Nikon CCD camera.

The time-resolved PL emission spectra and PL lifetime were measured on a streak camera (380–420 nm) using a femtosecond laser pulse from an optical parametric amplifier (Hamamatsu (Hamamatsu, Japan) Photonics C4780). The center wavelength of 360 nm was used as the excitation light source (Coherent Mira, Usho KEC-160).

Kuhn’s anisotropy factor in the ground state was defined as *g*_CD_ = 2×(*ε*_L_–*ε*_R_)/(*ε*_L_+*ε*_R_), where *ε*_L_ and *ε*_R_, respectively, denoted the molar absorptivity for left and right circularly polarized light. The magnitude of circular polarization in the excited states was defined as *g*_CPL_ = 2 × (*I*_L_–*I*_R_)/(* I*_L_+*I*_R_), where *I*_L_ and *I*_R_, respectively, stand for the signal intensities of left- and right-circularly polarized light under an unpolarized incident light beam. Experimentally, the value of g_CD_ was defined as *Δε*/*ε* = [Ellipticity/32,980]/Absorbance at a CD extremum, and for CPL amplitude, the value of *g*_CPL_ was defined as *ΔI*/*I* = [Ellipticity/(32,980/*ln*10)]/[Unpolarized PL intensity] at a CPL extremum [41,42].

### 3.3. Chemicals

Spectroscopic grade chloroform, THF, methanol, ethanol and isopropanol (Dotite) were used to prepare the polymer solutions and for measurements. (*R*)- and (*S*)-limonenes were obtained from Wako and purified by distillation under reduced pressure prior to use. (1*R*)-/(1*S*)-*α*-pinenes and **PF8** were purchased from Sigma–Aldrich and used as received. Our analysis showed that (*R*)-limonene: [*α*]^25^_589_ = +100.78° (neat), > 99.0% *ee*. (*S*)-limonene: [*α*]^25^_589_ = –100.97° (neat), > 99.0% *ee* [41,42]. Anthracene was purchased from TCI.

### 3.4. Preparation of PF8P2 Aggregates

The typical procedure for the production of **PF8P2** aggregates in a chloroform/(*R*)-limonene/ methanol tersolvent (0.3/1.1/1.6 (v/v/v)) was described below. To 0.3 mL of a chloroform solution containing **PF8P2** (1 × 10^−4^ M) in an SQ cuvette placed in a Peltier apparatus at 25 °C with CW stirring using a PTFE-coated cylindrical magnetic stir bar (2 mm in diameter and 4 mm in length), 1.1 mL of (*R*)-limonene was added. By further adding 1.6 mL of methanol to the solution, a white turbid **PF8P2** aggregate was formed. After stirring for ≈ 10–30 s, this solution was used for CD/UV-vis and CPL/PL measurements.

## 4. Conclusions

Limonene and *α*-pinene led to instant generation of optically active **PF8P2** aggregates from CD/CPL-silent **PF8P2** during aggregation with the help of good and poor solvents. (*S*)- and (*R*)-Limonenes provided optically active **PF8P2** aggregates with distinct CD/CPL amplitudes with a high *Φ*_PL_ of 16−20%, whose chiroptical signs are determined only by limonene chirality. In a series of experiments, several anomalous MSB effects can be seen. The CD- and/or CPL-amplitude and sign of the **PF8P2** aggregates depend on: (i) achiral solvent molecules (THF, chloroform, methanol, ethanol, isopropanol), (ii) chirality of limonene and *α*-pinene, (iii) CW and CCW stirring during aggregation (mechanophysical effect), (iv) addition of methanol to limonene-induced **PF8P2** aggregates. Several tersolvents, including chloroform/limonene/ethanol, chloroform/limonene/isopropanol, chloroform/ *α*-pinene/methanol, and THF/limonene/methanol, induce chiroptically detectable MSB effects.
